# Imaging large-scale cellular activity in spinal cord of freely behaving mice

**DOI:** 10.1038/ncomms11450

**Published:** 2016-04-28

**Authors:** Kohei J. Sekiguchi, Pavel Shekhtmeyster, Katharina Merten, Alexander Arena, Daniela Cook, Elizabeth Hoffman, Alexander Ngo, Axel Nimmerjahn

**Affiliations:** 1 Waitt Advanced Biophotonics Center, The Salk Institute for Biological Studies, La Jolla, California 92037, USA

## Abstract

Sensory information from mechanoreceptors and nociceptors in the skin plays key roles in adaptive and protective motor behaviours. To date, very little is known about how this information is encoded by spinal cord cell types and their activity patterns, particularly under freely behaving conditions. To enable stable measurement of neuronal and glial cell activity in behaving mice, we have developed fluorescence imaging approaches based on two- and miniaturized one-photon microscopy. We show that distinct cutaneous stimuli activate overlapping ensembles of dorsal horn neurons, and that stimulus type and intensity is encoded at the single-cell level. In contrast, astrocytes show large-scale coordinated calcium responses to intense but not weak sensory inputs. Sensory-evoked activity is potently suppressed by anaesthesia. By revealing the cellular and computational logic of spinal cord networks under behaving conditions, our approach holds promise for better understanding of healthy and aberrant spinal cord processes.

## Abstract (web summary)

Imaging cellular activity in mouse spinal cord has been historically difficult. Here the authors develop cellular resolution fluorescence imaging approaches in the spinal cord of behaving mice, and report distinct activity patterns of neurons and astrocytes in response to different sensory inputs.

## Graphical abstract

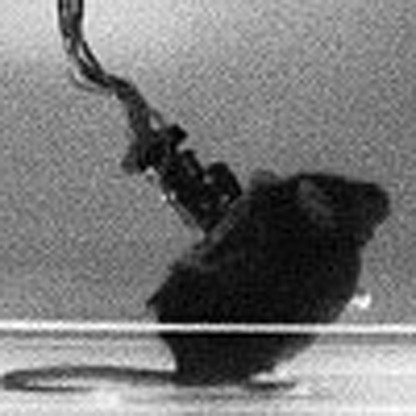

Deciphering how cellular activity in the central nervous system encodes sensory information and animal behaviour remains one of the greatest challenges in neuroscience research today. Traditionally, electrophysiological approaches have been used to sparsely sample from electrically excitable cells. Optical imaging in combination with new labelling approaches now allows minimally invasive and comprehensive sampling from dense networks of both electrically and chemically excitable cells, such as neurons and glial cells^1^. Imaging in head-restrained mobile mice and with miniaturized head-borne microscopes, for example, has led to the discovery of unanticipated forms of behaviourally related neuronal and astrocyte excitation in cortical and deep brain microcircuits^2^,^3^,^4^,^5^. Application of these imaging modalities to the spinal cord, the primary neurological link between the brain and other parts of the body, promises to yield new insight into how genetically defined cell types and their activity patterns contribute to spinal cord physiology and animal behaviour. In particular, given its ordered cellular arrangement, the spinal cord presents unique opportunities for studying neuron–glia communication and its role in central nervous system function^6^,^7^.

Spinal dorsal horn circuits are involved in sensory information processing, including pain perception^8^,^9^. How neuronal components that make up these circuits encode information coming from the skin or deeper tissues is unclear. Likewise, dorsal horn astrocytes structurally and functionally interact with spinal neurons and contribute to pain sensation^10^,^11^,^12^. How astrocyte activity relates to spinal neuron spiking, and to what extent these cells encode sensory information in behaving mice is unknown.

To enable stable measurement of neuronal and astrocyte activity in the spinal cord of behaving mice, we further developed fluorescence imaging approaches based on two- and miniaturized one-photon microscopy and, by comparing the two modalities in the same animal, we provide practical guidelines for their future use in the central nervous system. We demonstrate that distinct cutaneous inputs in awake mice activate partially overlapping subsets of neurons, and that type and intensity of sensory information is encoded at the single-neuron level. In contrast, sensory stimulus parameters appeared only weakly reflected in individual calcium transients from dorsal horn astrocytes. Astrocyte population activity, including large-scale coordinated calcium transients in response to high- but not low-intensity sensory stimulation, correlated with certain aspects of sensory input. General anaesthesia potently suppressed calcium activity in both dorsal horn neurons and astrocytes.

## Results

### Fluorescence imaging in spinal cord of freely behaving mice

**Figure 1:**
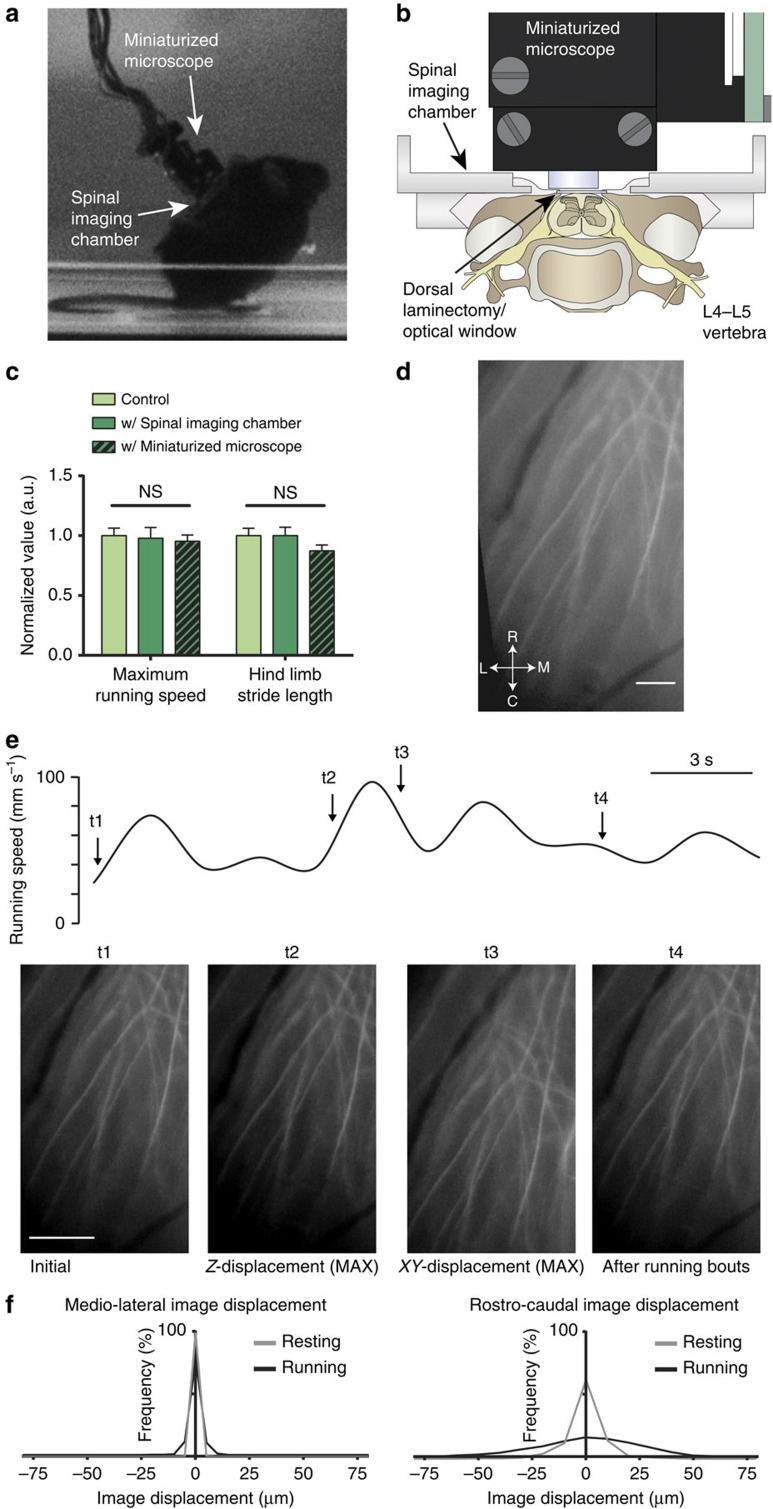
Cellular resolution imaging in spinal cord of freely behaving mice. (**a**) Image of a mouse with a custom miniaturized one-photon microscope mounted over a spinal imaging chamber implanted at the lumbar level. Mouse behaviour on a motorized linear treadmill was videotaped. Video and imaging data were synchronized before analysis. (**b**) Schematic showing the implanted imaging chamber and mounted miniaturized one-photon microscope in cross-section. The chamber was implanted at the lumbar level. (**c**) Population data showing maximum running speed and hind limb stride length on the motorized linear treadmill for mice implanted with a spinal imaging chamber (dark green), the same mice with a miniaturized microscope mounted over the imaging chamber (patterned dark green) or the same mice before the surgery (light green). Behavioural measurements of mice with a spinal imaging chamber were taken 1 day after implantation. (**d**) Fluorescence image showing yellow fluorescent protein-expressing axons in dorsal spinal cord of a Thy1-eYFP-H mouse acquired with the miniaturized one-photon microscope before terminating anaesthesia. The subregion image is an average of 800 frames acquired at 45 f.p.s. Medio-lateral and rostro-caudal direction is indicated. Scale bar, 50 μm. (**e**) Top: mouse treadmill running speed (black trace) over a 20-s recording period. Bottom: subregion images showing the initial frame (t1) and frames with maximum image displacement (t2 and t3) during the awake recording period. Image displacement was transitory (t4) and mainly occurred in rostro-caudal direction. Scale bars, 3 s (top); 100 μm (bottom). (**f**) Population data showing probability distribution of medio-lateral (left) and rostro-caudal (right) image displacements during rest (grey) and running (black). Dorso-ventral image displacements were ≤5 μm (see Methods). Data are represented as mean±s.e.m. (**c**) Comparison between the same mice before and after surgery; one-way repeated measures ANOVA (*P*≥0.05; *N*=5 mice per group).

As a first step towards optical recording of cellular dynamics in the spinal cord of behaving mice, we created custom miniaturized one-photon microscopes for high-speed fluorescence imaging with subcellular resolution, similar to our previous work^3^,^5^ (Fig. 1a). We established a spinal cord window preparation for *in vivo* imaging of spinal dorsal horn^13^, and for stable attachment of our 2.5-g miniaturized microscope to the animal's back (Fig. 1a,b). Before and 1 day after surgical preparation, we quantified the animal's locomotor performance on a motorized linear treadmill. Adult wild-type mice implanted with a lumbar spinal cord window and an attached miniaturized microscope showed no overt signs of altered locomotor behaviour, even without habituation. Maximum running speed and hind limb stride length values were comparable to the same mice before surgical preparation (Fig. 1c; one-way repeated measures analysis of variance (ANOVA); *P*≥0.05; *N*=5 mice per group).

Next, to quantify lateral and axial motion artefacts during imaging, we implanted spinal cord windows in transgenic Thy1-eYFP-H mice with sparsely labelled axons (Fig. 1d)^14^. In awake resting mice, image displacements due to breathing or minor body movements occurred primarily in the rostro-caudal direction (mean±s.e.m., 8.5±8.0 μm; 75% percentile within 11.2 μm; *N*=4 mice), while displacements in the medio-lateral (1.5±0.3 μm; 75% percentile within 2.1 μm; *N*=4 mice) or dorso-ventral direction (≤3 μm; *N*=4 mice) were small. Motion artefacts increased in amplitude during steady walking or running but remained largely confined to the rostro-caudal direction (22.3±16.8 μm; 75% percentile within 29.1 μm; *N*=4 mice) with only small increases in medio-lateral (3.2±1.6 μm; 75% percentile within 3.9 μm; *N*=4 mice) or dorso-ventral image displacement (≤5 μm; *N*=4 mice; Fig. 1e,f), allowing continuous video-rate tracking of cells within central regions of the 350 μm × 700 μm effective field of view (FOV; Supplementary Movies 1, 2). Image displacements were largely uniform across the FOV, allowing automated or manual subpixel registration^15^. During certain movements, such as grooming or left/right side bending of the spinal cord (for example, when the animal made a sharp turn), image displacements across the FOV were non-uniform, typically increasing with distance from the central vein. We restricted our motion artefact analysis to dorsal horn regions.

To determine attainable imaging depth with our miniaturized one-photon microscopes, we made use of transgenic mice expressing tdTomato in genetically defined dorsal horn neurons with known laminar distribution^16^,^17^ (Supplementary Fig. 1a). Imaging the same dorsal horn region with two-photon and miniaturized one-photon microscopy in anaesthetized mice with labelled somatostatin lineage neurons (SOM-tdTomato mice) revealed that cells located as deep as outer lamina II are readily accessible with both imaging modalities (Supplementary Fig. 1b–c). This enables optical monitoring of cellular circuits involved in sensory or pain processing^8^,^9^. The extended depth of field of one- as compared with two-photon microscopy allowed concomitant monitoring of cells located at different depth (Supplementary Fig. 1b–c) and, for a given image sensor frame acquisition rate, across a larger FOV than point scanning-based two-photon microscopy.

### Fluorescence imaging in spinal cord of awake mobile mice

**Figure 2:**
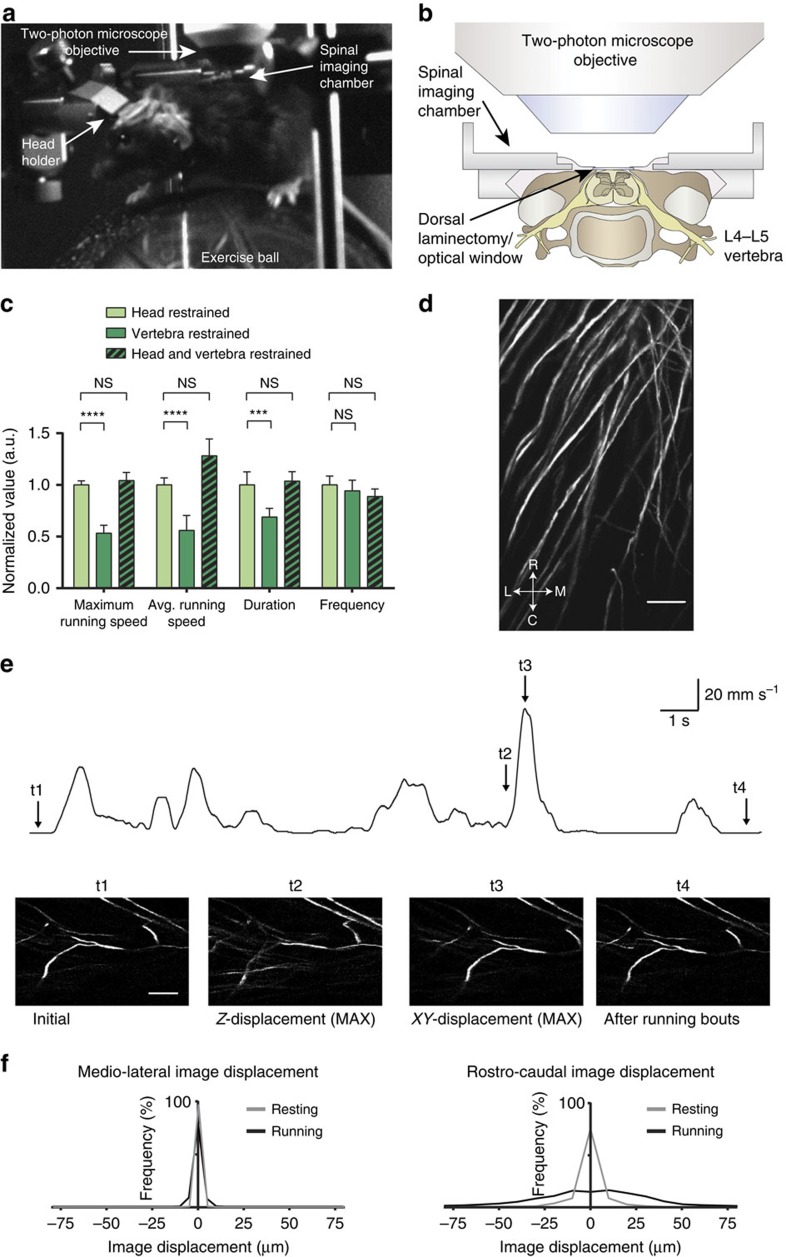
Cellular resolution imaging in spinal cord of awake mobile mice. (**a**) Image of a mouse focally restrained at the level of the spinal cord and head, and placed on a spherical treadmill under a two-photon microscope. (**b**) Schematic showing the implanted imaging chamber and two-photon microscope objective in cross-section. The chamber was implanted at the L4–L5 vertebra level. (**c**) Population data showing maximum and average running speed, and average running duration and frequency on the spherical treadmill for mice restrained at the lumbar vertebra level (dark green), the same mice restrained at the lumbar vertebra level and head (patterned dark green) or only at the head (light green). Measurements under the three restrained conditions were randomized. Behavioural measurements were taken 7 days after chamber implantation and after 5 days of habituation. (**d**) Fluorescence image showing yellow fluorescent protein-expressing axons in dorsal spinal cord of the same Thy1-eYFP-H mouse shown in Fig. 1d, but acquired with a two-photon microscope. The image is a maximum intensity projection through a stack of 25 images acquired under anaesthesia (axial spacing, 1  μm; frame average, 8; frame rate, 30.9 f.p.s.). Medio-lateral and rostro-caudal direction is indicated. Scale bar, 50 μm. (**e**) Top: mouse treadmill running speed (black trace) during a 20-s recording period. Bottom: images showing the initial frame (t1) and frames with maximum image displacement (t2 and t3) during the awake recording period. Image displacement was transitory (t4) and occurred mainly in rostro-caudal direction. Scale bars, 20 mm s^−1^ and 1 s (top); 50 μm (bottom). (**f**) Population data showing probability distribution of medio-lateral (left) and rostro-caudal (right) image displacements during rest (grey) and running (black). Axial displacements were typically ≤5 μm (see Methods). Mice were habituated to spinal cord and head restraint for typically 5 days before optical measurements. Data are represented as mean±s.e.m. (**c**) Comparison between mice under different restrained conditions; one-way repeated measures ANOVA, *N*=6 mice per group (*P*<0.05 for comparison between ‘Head-restrained' and ‘Vertebra-restrained' groups under ‘Maximum running speed', ‘Avg. running speed' and ‘Duration'; *P*≥0.05 for all other comparisons). Avg., average.

When imaging small-scale structures such as single axons, two-photon microscopy provided superior contrast and spatial resolution (Supplementary Fig. 2), and optical access to dorsal horn regions below outer lamina II. Given these particular advantages, we sought to implement approaches for two-photon imaging in spinal cord of awake mobile mice (Fig. 2a,b). Restraining mice at the level of the implanted spinal cord chamber, analogous to head-restrained brain imaging^2^,^4^, resulted in reduced maximum and average running speed of mice on a spherical treadmill (Fig. 2c; one-way repeated measures ANOVA; *P*<0.05; *N*=5 mice per group). Likewise, duration of running was reduced compared with head-restrained mice (Fig. 2c; one-way repeated measures ANOVA; *P*<0.05; *N*=6 mice per group). However, when we restrained mice both at the level of the spinal cord and head, using implanted chambers on either site, mice showed locomotor performance comparable to head-restrained mice (Fig. 2c; one-way repeated measures ANOVA; *P*≥0.05; *N*=6 mice per group). The additional head restraint prevented mice from left/right bending of their upper body and further reduced motion artefacts.

To quantify lateral and axial motion artefacts during two-photon imaging in awake mobile mice, we performed video-rate optical recordings in transgenic Thy1-eYFP-H mice (Fig. 2d). As in the case of freely moving mice, image displacements occurred primarily in the rostro-caudal direction during awake resting and running periods (7.5±7.1 μm, 75% percentile within 10.5 μm, and 21.9±17.3 μm, 75% percentile within 29.9 μm, respectively; *N*=3 mice; Fig. 2e,f). Medio-lateral and dorso-ventral image displacements were generally small in both awake resting (1.5±0.2 μm, 75% percentile within 2.0 μm, and ≤3 μm, respectively; *N*=3 mice) and running mice (3.0±0.9 μm, 75% percentile within 4.0 μm, and ≤5 μm, respectively; *N*=3 mice; Fig. 2e,f), allowing continuous tracking of cells at 30.9 f.p.s. for most behaviours within central regions of the 270 μm × 360 μm effective FOV without adaptive focus control^18^ (Supplementary Movie 3).

### Sensory information encoding by dorsal horn neurons

**Figure 3:**
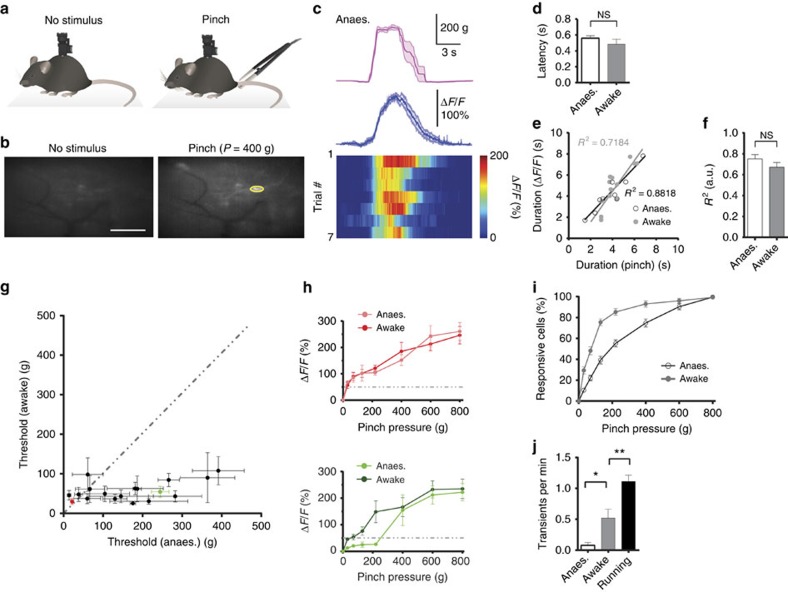
Dorsal horn neurons encode sensory information at the single-cell level. (**a**) Schematic of experimental approach. Calcium spiking in dorsal horn neurons of anaesthetized and awake mice injected with AAV9-CaMKII-GCaMP6f was recorded during rest (left) and following cutaneous stimulation with a rodent pincher applied to the base of the animal's tail (right). (**b**) Example fluorescence images showing transduced cells during rest (left) and in response to tail pinch (right; *P*=400 g) under anaesthesia. A somatic region of interest (ROI) is indicated (yellow). Scale bar, 100 μm. (**c**) Pinch-evoked calcium responses in the ROI highlighted in **b**. Top: average trace of applied pinch stimulus. Centre: average pinch-evoked calcium transient. Bottom: individual calcium transients for seven individual trials. (**d**) Population data showing calcium transient latency with respect to pinch onset for anaesthetized (white) and awake mice (grey; see Methods). (**e**) Relationship between pinch and calcium transient duration for a representative dorsal horn neuron under anaesthetized (open circles) and awake conditions (closed circles; *P*=220 g). A linear fit to the anaesthetized and awake data (black and grey line, respectively) is shown. (**f**) Population data showing that calcium responses in dorsal horn neurons linearly encoded pinch duration in anaesthetized and awake mice. Analysis included only cells that responded under both anaesthetized and awake conditions. (**g**) Population data showing the responsiveness of individual dorsal horn neurons under anaesthetized and awake conditions. (**h**) Relationship between pinch pressure and calcium transient amplitude for the two example neurons indicated in **g** (red and green). Dotted horizontal line indicates calcium transient amplitude (50% Δ*F*/*F*) below which cells were considered unresponsive. (**i**) Population data showing that for a given pinch pressure less neurons responded under anaesthetized (open circles) compared with awake conditions (closed circles). (**j**) Population data showing calcium transient frequency in dorsal horn neurons under anaesthetized (white), awake resting (grey) and running conditions (black). Data are represented as mean±s.e.m. (**d**) Unpaired *t*-test with Welch's correction, *n*=43 cells for ‘Anaes.' and *n*=59 cells for ‘Awake' in *N*=4 mice per group, *P*≥0.05. (**f**) Unpaired *t*-test with Welch's correction, *n*=38 cells in *N*=4 mice per group, *P*≥0.05. (**j**) Unpaired *t*-test with Welch's correction, *n*=28 cells in *N*=4 mice per group, *P*<0.05. Anaes., anaesthetized.

Having established and compared two- and miniaturized one-photon imaging in spinal cord of behaving mice, we proceeded with functional measurements based on the changes in intracellular calcium concentration. To monitor calcium activity in dorsal horn neurons, we injected AAV9-CaMKII-GCaMP6f at the lumbar (L4–L5 vertebra) level 14 days before spinal cord window implantation (Supplementary Fig. 3a–d). Calcium imaging using miniaturized one-photon microscopy was performed immediately after spinal cord window implantation. In anaesthetized mice, a subset of fluorescently labelled neurons across the FOV reliably responded to focal pinch to the base of the animal's tail with a latency of ∼0.5 s (Fig. 3a–d; Supplementary Movie 1; Methods). Tail pinch with calibrated forceps (see Methods) was chosen because it can be readily and reproducibly applied in awake mice^19^. Focal pinch to the animal's hind paws elicited little or no discernible calcium transients.

To gain deeper insight into how dorsal horn neurons encode pinch, we systematically varied pinch duration while keeping other stimulus parameters such as pressure amplitude or position constant (Fig. 3e). Using a cell-sorting algorithm based on principal and independent component analysis (PCA/ICA)^20^ supplemented by manual region of interest (ROI) analysis (see Methods), population calcium imaging data from 37 cells in four mice revealed a linear relationship between pinch and calcium transient duration (*R*^2^=0.75±0.04; *n*=37 cells in *N*=4 mice; Fig. 3f). When we varied pinch pressure, while keeping pinch duration and position constant, individual neurons' calcium transient amplitude showed a steady but non-linear increase (Fig. 3g,h). Two-photon measurement of the same cells confirmed these findings (Supplementary Fig. 4). We also found that the number of activated dorsal horn neurons increased with pinch pressure (Fig. 3i).

Next, we asked how cellular response properties differ between anaesthetized and awake mice. Repeating above pinch experiments in the same mice after they had recovered from isoflurane anaesthesia for at least 1.5 h, we found that the calcium transient-to-stimulus duration relationship still showed a largely linear relationship (*R*^2^=0.67±0.05; *n*=68 cells in *N*=4 mice; Fig. 3e,f). However, a larger number of neurons responded to a given pinch pressure (Fig. 3g–i). Closer examination of pinch-evoked responses of individual neurons under anaesthetized and awake conditions revealed that general anaesthesia decreased responsiveness of dorsal horn neurons (Fig. 3h,i).

To test whether dorsal horn neural activity may be modulated by behavioural state, we tracked animals' behaviour in their home cage using video recordings. In awake resting mice, spontaneous firing of labelled dorsal horn neurons was low but increased compared with the same mice under anaesthesia (0.52±0.15 and 0.08±0.04 Hz, respectively; *n*=28 cells in *N*=4 mice; paired *t*-test with Welch's correction; *P*<0.05; Fig. 3j). Locomotor activity further increased neuronal firing rate (1.10±0.11 Hz; *n*=28 cells in *N*=4 mice; paired *t*-test with Welch's correction; *P*<0.05; Fig. 3j), but did not evoke spatiotemporal patterns of neural activity seen in response to pinch (Supplementary Movie 1). Pinch-evoked calcium transients reliably occurred at pinch onset and their duration was tightly correlated with stimulus duration (Fig. 3c–f).

**Figure 4:**
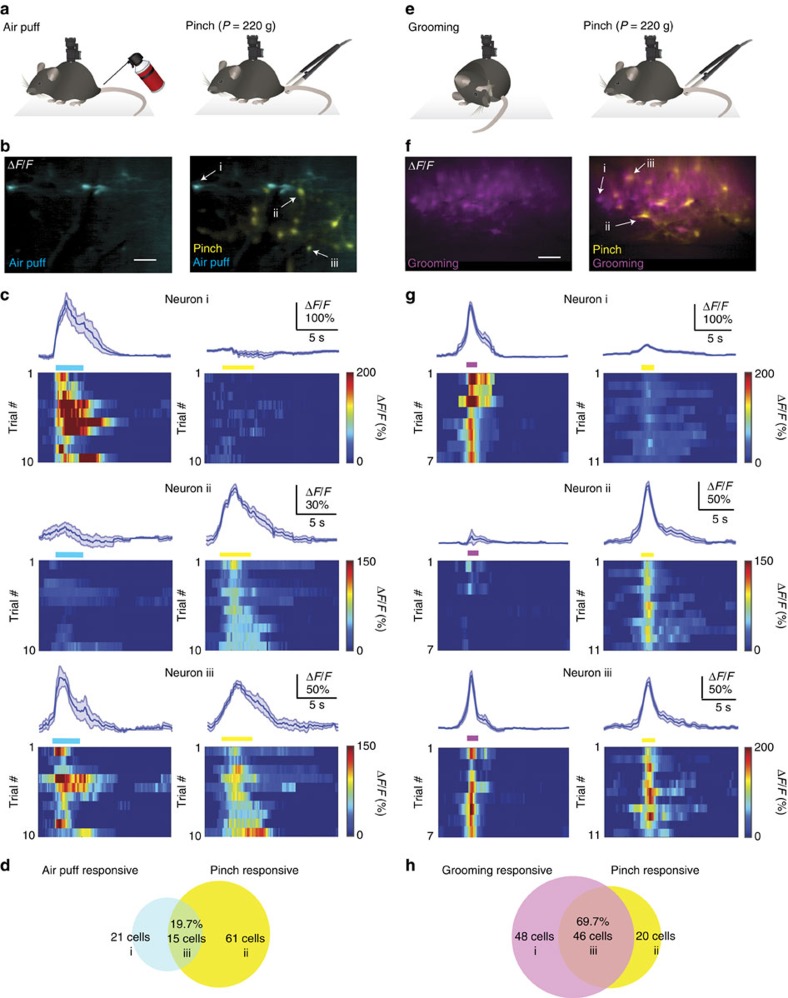
Distinct cutaneous inputs recruit different subsets of dorsal horn neurons. (**a**) Schematic of experimental approach. Two different cutaneous stimuli (pinch and air puff) were applied to the base of the animal's tail while monitoring calcium spiking of dorsal horn neurons in wild-type mice injected with AAV9-CaMKII-GCaMP6f at the L4–L5 vertebra level under unrestrained awake conditions using a miniaturized microscope. (**b**) Example fluorescence images showing dorsal horn neurons activated by air puff (blue) or pinch (yellow). Overlay is shown on right. Note their different spatial distribution. Three example neurons are indicated. Scale bar, 100 μm. (**c**) Average calcium transient (blue trace, top) and single-trial responses (bottom) to air puff (left) or pinch (right) for the three example neurons indicated in **b**. Blue and yellow horizontal bars indicate peripheral stimulus duration. Neuron i responded to air puff only, neuron ii to pinch only and neuron iii to air puff and pinch. (**d**) Population data showing the number of dorsal horn neurons within the field of view responding to air puff only, pinch only or pinch and air puff. Around 20% of pinch-responsive cells also responded to air puff. (**e**) Peripherally evoked calcium spiking in unrestrained awake mice (right) was compared with self-initiated hindquarter grooming near the base of the animal's tail (left). (**f**) Example fluorescence images showing dorsal horn neurons activated by hindquarter grooming (purple) or pinch (yellow). Overlay is shown on right. Note their different spatial distribution. Three example neurons are indicated. Scale bar, 100 μm. (**g**) Average calcium transient (blue trace, top) and single-trial responses (bottom) to hindquarter grooming (left) or pinch (right) for the three neurons indicated in **f**. Purple and yellow horizontal bars indicate stimulus duration. Neuron i responded to grooming only, neuron ii to pinch only and neuron iii to grooming and pinch. (**h**) Population data showing the number of dorsal horn neurons within the field of view responding to grooming only, pinch only or pinch and grooming. Around 70% of pinch-responsive cells also responded to grooming. Data are represented as mean±s.e.m.

Having characterized the response properties of dorsal horn neurons to pinch, we next asked how the same neurons (*n*=211 cells in *N*=3 mice) respond to different types of cutaneous stimuli. When we applied an air puff to the base of the animal's tail under anaesthetized or awake conditions, a distinct subset of labelled dorsal horn neurons responded reliably (Fig. 4a,b). Some neurons preferentially responded to air puff (*n*=21 cells in *N*=3 mice), while others responded to pinch only (*n*=61 cells in *N*=3 mice; Fig. 4c; Supplementary Fig. 5). Around 20% of pinch-responsive neurons responded reliably to air puff (*n*=15 cells in *N*=3 mice; 25 air puffs; Fig. 4d). When mice groomed their hindquarters, near the base of their tail, a large subset of pinch-responsive neurons (69.7%; *n*=46 cells in *N*=2 mice; 14 grooming events) responded with calcium transients (Fig. 4e–h; Supplementary Movie 2). No such response was seen when mice engaged in forequarter grooming. In contrast to ‘pinch'- and ‘air puff'-responsive cells, neurons that were activated by self-initiated hindquarter grooming appeared more evenly distributed throughout dorsal horn at the L4–L5 vertebra level (Fig. 4b,f). These results demonstrate that distinct cutaneous stimuli to the same bodily region can recruit different, partially overlapping ensembles of dorsal horn neurons.

### Sensory information encoding by dorsal horn astrocytes

Dorsal horn neurons are embedded in a dense network of spinal cord astrocytes. Astrocytes structurally and functionally interact with neurons in spinal cord and brain. Astrocytes show neuronal activity-dependent forms of calcium excitation. In cortical regions of behaving mice, different spatiotemporal forms of astrocyte calcium activity have been identified^21^. However, it is unknown what forms of astrocyte calcium activity exist in the spinal cord, and how these transients relate to calcium spiking in spinal neurons of behaving mice. To begin to address this question, we transduced spinal cord astrocytes at the L4–L5 vertebra level with AAV5-GfaABC1D-GCaMP6f 21 days before spinal cord window preparation (Supplementary Fig. 3e–f). Imaging was performed on the day of window implantation. While miniaturized one-photon microscopy allowed large dorsal horn areas to be monitored in freely moving mice, its limited spatial resolution and the dense labelling of astrocytes and their fine processes hampered reliable detection of astrocyte microdomain activations. To monitor calcium activity in astrocyte fine processes and somata of lamina I and II, we performed two-photon imaging in awake mice on a spherical treadmill, restrained at the spinal cord and head level, and after they had recovered from isoflurane anaesthesia for at least 1.5 h.

**Figure 5:**
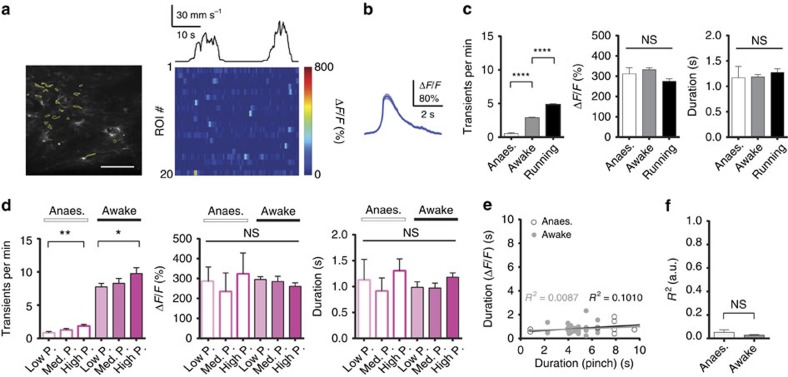
Small-scale, unsynchronized calcium transients in dorsal horn astrocytes weakly correlate with sensory information. (**a**) Left: example fluorescence image showing dorsal horn astrocytes at ∼30-μm depth in wild-type mice injected with AAV5-GfaABC1D-GCaMP6f. Select astrocyte process regions of interest (ROIs) are indicated (yellow). Right: calcium activity traces of the indicated ROIs during a 45-s recording period in an awake mouse. Mouse running speed (black) is shown on top. Running did not evoke large-scale coordinated calcium excitation. Scale bar, 100 μm. (**b**) Average time course of unsynchronized calcium transients in astrocyte processes. Shown trace is an average of *n*=1065 events in *N*=4 mice. (**c**) Population data showing calcium transient frequency (left), amplitude (centre) and duration (right) of unsynchronized astrocyte calcium transients in anaesthetized (white), awake resting (grey) and running mice (black). (**d**) Population data from individual astrocyte processes showing unsynchronized calcium transient frequency (left), amplitude (centre) and duration (right) in response to low- (pink; *P*=30–70 g), medium- (magenta; *P*=130–220 g) or high-amplitude pinch (purple; *P*≥400 g) in anaesthetized (open bars) or awake mice (solid bars). (**e**) Relationship between pinch and astrocyte calcium transient duration for a representative astrocyte process ROI under anaesthetized (open circles) and awake conditions (closed circles; *P*=220 g). A linear fit to the anaesthetized and awake data (black and grey line, respectively) is shown. (**f**) Population data showing that individual astrocyte calcium transients do not encode pinch duration in anaesthetized or awake mice. Data are represented as mean±s.e.m. (**c**) Left panel shows unpaired *t*-test, *n*=84 ROIs in *N*=4 mice per group, *P*<0.05; centre and right panels show one-way ANOVA with Tukey–Kramer's multiple comparisons test (*P*<0.05; *n*=84 ROIs in *N*=4 mice per group). (**d**) Left panel shows unpaired *t*-test, *n*=84 ROIs in *N*=4 mice, *P*<0.05; centre and right panels show one-way ANOVA with Tukey–Kramer's multiple comparisons test (*P*≥0.05; *n*=84 ROIs in *N*=4 mice). (**f**) Unpaired *t*-test, *n*=37 ROIs for ‘Anaes.' and *n*=42 ROIs for ‘Awake' in *N*=4 mice, *P*≥0.05. Anaes., anaesthetized; med., medium.

In awake resting mice, calcium transient frequency in processes of dorsal horn astrocytes was low but elevated compared with the same mice under anaesthesia (2.91±0.06 transients per min and 0.53±0.12 transients per min, respectively; *n*=84 ROIs in *N*=4 mice; paired *t*-test with Welch's correction; *P*<0.05; Fig. 5a–c). Process activity was further increased in running animals (4.83±0.15 transients per min; *n*=84 ROIs in *N*=4 mice; Fig. 5a–c). Unlike in the cortex^4^,^22^,^23^, running alone did not evoke large-scale coordinated calcium events (Fig. 5a). Amplitude and duration of calcium transients in astrocyte processes were comparable between anaesthetized, awake resting and running mice (Fig. 5c; one-way ANOVA with Tukey–Kramer's multiple comparisons test; P≥0.05; *n*=84 ROIs in *N*=4 mice per group).

**Figure 6:**
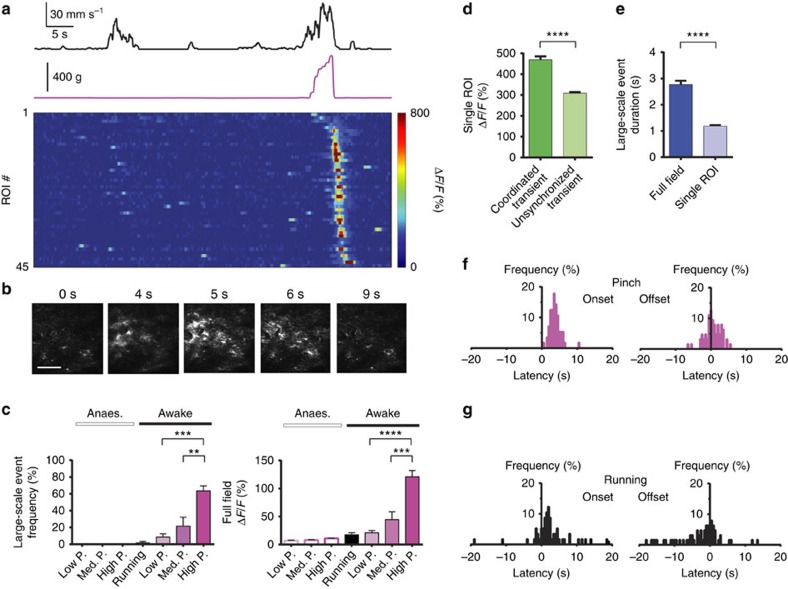
Dorsal horn astrocytes show large-scale coordinated calcium excitation to strong but not weak cutaneous input. (**a**) Calcium activity traces in 45 astrocyte ROIs during a 67-s recording period in an awake mouse. Mouse running speed (black) and tail pinch application (purple) is shown on top. (**b**) Example fluorescence images showing the high-amplitude pinch-evoked coordinated astrocyte calcium excitation of **a**. Time after pinch onset is indicated. Scale bar, 100 μm. (**c**) Population data showing occurrence frequency (left) of large-scale coordinated calcium events, and average full-field calcium transient amplitude (right), in anaesthetized (open bars) or awake mice (solid bars), and in response to low- (pink), medium- (magenta) or high-amplitude pinch (purple). (**d**) Population data showing that the average calcium transient amplitude in individual astrocyte ROIs is larger during large-scale coordinated events compared with unsynchronized calcium transients. (**e**) Population data showing that the duration of large-scale events within the field of view is longer than the duration of individual transients contributing to large-scale events. (**f**) Population data showing latency of large-scale astrocyte calcium events with respect to pinch onset (left) or offset (right). Large-scale events are triggered by pinch onset and have a latency of ∼4 s. (**g**) Population data showing latency of large-scale events with respect to the temporally nearest running onset (left) or offset (right). Large-scale events are not triggered by running alone. Data are represented as mean±s.e.m. (**c**) Left panel shows paired *t*-test with Welch's correction, *n*=38, 39, 61, 48, 37, 44 or 97 trials (from left to right) in *N*=4 mice per group, *P*<0.05; right panel shows unpaired *t*-test with Welch's correction; *n*=38, 39, 61, 48, 37, 44 or 97 trials (from left to right) in *N*=4 mice per group, *P*<0.05. (**d**) Unpaired *t*-test with Welch's correction, *n*=71 ROIs in *N*=4 mice per group, *P*<0.05. (**e**) Unpaired *t*-test, *n*=56 for ‘Full field' and *n*=71 for ‘Single ROI' in *N*=4 mice per group, *P*<0.05. Anaes., anaesthetized; med., medium.

Low- (*P*=30–70 g) or medium-amplitude (*P*=130–220 g) pinch to the base of the animal's tail led to a modest increase in the frequency of astrocyte process activations, but no significant change in calcium transient amplitude or duration in anaesthetized (0.85±0.19 transients per min, 286±72% Δ*F*/*F*, 1.13±0.40 s or 1.28±0.20 transients per min, 235±93% Δ*F*/*F*, 0.92±0.25 s, respectively) or awake mice (7.75±0.52 transients per min, 295±14% Δ*F*/*F*, 0.98±0.11 s or 8.29±0.71 transients per min, 261±17% Δ*F*/*F*, 0.97±0.10 s, respectively; unpaired *t*-test with Welch's correction; *P*<0.05; one-way ANOVA with Tukey–Kramer's multiple comparisons test; *P*≥0.05; *n*=84 ROIs in *N*=4 mice; Fig. 5d). Unlike calcium spiking in dorsal horn neurons, astrocyte calcium transients that occurred during pinch had variable delay with respect to stimulus onset, and their duration did not significantly increase with pinch duration in anaesthetized or awake mice (*R*^2^=0.05±0.02 or *R*^2^=0.03±0.01, respectively; *n*=37 ROIs in *N*=4 mice; Fig. 5e,f). However, when we increased pinch pressure, we found that high-amplitude (*P*=400–800 g) stimuli evoked, with high probability (63.9±5.9%; *n*=97 stimuli in *N*=4 mice), large-scale coordinated calcium events in astrocyte processes and somata across the FOV with an onset latency of ∼3.75±0.22 s (*n*=62 events in *N*=4 mice; Fig. 6a–f; Supplementary Movie 3; Methods). The average amplitude of individual calcium transients within large-scale events was larger than that of unsynchronized transients in astrocyte processes (469±16% Δ*F*/*F* and 308±6% Δ*F*/*F*, respectively; unpaired *t*-test with Welch's correction; *n*=71 ROIs in *N*=4 mice; *P*<0.05; Fig. 6d). The duration of the coordinated population event (2.77±0.15 s; *n*=62 events in *N*=4 mice) was longer than the duration of individual transients contributing to the large-scale event (1.19±0.03 s; unpaired *t*-test; *n*=71 ROIs in *N*=4 mice; *P*<0.05; Fig. 6e).

High-amplitude pinch stimuli tended to evoke locomotor activity (94.8±2.6% of P≥400 g stimuli; maximum running speed, 80.3±3.6 mm s^−1^; *n*=97 stimuli in *N*=4 mice). However, the latency of large-scale calcium transients in astrocytes was more tightly correlated with pinch onset than with locomotor activity (Fig. 6f,g). No correlation was found with either pinch offset or running offset (Fig. 6f,g). Locomotor activity alone did not evoke large-scale calcium events (Figs 5a; Fig. 6a,c). Low- or medium-amplitude pinch stimuli also tended to evoke locomotor activity (74.9±8.8% or 83.2±7.7% of *P*=30–70 g or *P*=130–220 g stimuli, respectively; *n*=36 or 44 stimuli, respectively, in *N*=4 mice) with similar characteristics (maximum running speed, 71.4±5.2 mm s^−1^ or 77.0±5.8 mm s^−1^, respectively; Kruskal–Wallis test; *n*=36 or 44 stimuli, respectively, in *N*=4 mice; *P*≥0.05), yet large-scale events occurred significantly less often under these conditions (8.6±3.8% or 21.6±10.5% probability, respectively; *n*=36 or 44 stimuli, respectively, in *N*=4 mice). No large-scale calcium transients were seen under general anaesthesia (Fig. 6c). Large-scale activity occurred bilaterally and over at least 1 mm in rostro-caudal direction, as revealed by two- and miniaturized one-photon microscopy. Air puff stimulation near the base of the animal's tail was also capable of evoking large-scale astrocyte calcium transients (40% probability; *n*=10 stimuli in *N*=3 mice). Together, astrocytes in dorsal horn of behaving mice showed spatiotemporal activity patterns and threshold responses remarkably different from those of dorsal horn neurons.

## Discussion

We demonstrate stable imaging of sensory-evoked cellular activity in spinal cord of freely behaving mice using miniaturized one-photon microscopy. By comparing one- and two-photon recordings of the same cells in the same animal, we provide practical guidelines for future use of the two imaging modalities in behaving mice. We demonstrate that distinct cutaneous stimuli recruit different, overlapping ensembles of dorsal horn neurons. Evoked single cell and population activity in dorsal horn astrocytes showed remarkable differences to dorsal horn neurons, including large-scale coordinated calcium events in response to high- but not low-intensity peripheral stimuli. Unlike grey matter astrocytes of the brain^4^,^22^,^23^, dorsal horn astrocytes did not show large-scale coordinated calcium events triggered by locomotor activity alone. Spinal neuron and astrocyte calcium activity was potently suppressed by general anaesthesia.

Miniaturized one-photon microscopy enables stable high-speed optical recording of cellular activity from superficial and deep brain regions in freely moving mice^3^,^5^,^24^,^25^,^26^. We demonstrate that this approach also enables stable cellular activity measurements from spinal cord of freely behaving mice, despite the more pronounced movement of the spine within the vertebral column compared with the brain within the skull (Figs 1f and 2f)^2^,^4^. Lateral and axial motion artefacts during optical recordings were comparable between focally restrained and freely moving mice (Figs 1f and 2f). For animal behaviours associated with large axial movements, including natural behaviours non-executable in restrained mice, volume imaging with vertebra-affixed miniaturized one-photon microscopes offered greater recording stability compared with optical section acquisition with multi-photon microscopy. Camera-based one-photon imaging also allowed monitoring of larger spinal cord areas, or at higher frame rate, compared with point scanning-based two-photon microscopy. The curvature of the spinal cord constrained the number of cells that could be monitored laterally.

Previous studies in anaesthetized rodents, using two-photon imaging of non-selective synthetic or genetically encoded calcium indicators, showed that electrical or mechanical peripheral stimulation can evoke calcium spiking in subsets of dorsal horn neurons, with the number of activated neurons depending on stimulus location and intensity^18^,^27^,^28^,^29^. In addition, using *in utero* electroporation of CAG promoter-driven YC-Nano50, Nishida *et al*.^29^ found that pinch, brush or heat activates partially overlapping subsets of dorsal horn neurons in anaesthetized mice.

We demonstrate that stimulus-evoked calcium spiking in dorsal horn neurons is potently suppressed by general anaesthesia (Fig. 3g–j), and that in awake mice calcium spiking is modulated by behavioural state (Fig. 3j), akin to cortical findings^2^,^20^,^30^. By systematically varying pinch parameters, we show that stimulus properties are encoded at the single-neuron level (Figs 3 and 4). In addition, we found that different types of peripheral stimuli, including tail pinch, air puff and hindquarter grooming, activated overlapping ensembles of dorsal horn neurons (Fig. 4b–d,f–h). While pinch involves activation of mechanoreceptors, air puff and grooming may involve activation of multiple sensory modalities. Future studies in behaving mice are needed to determine how activation of individual sensory or descending pathways contributes to the described calcium responses in AAV9-CaMKII-GCaMP6f-transduced cells. Future combined imaging and electrophysiology experiments are needed to address how calcium transients in transduced neurons relate to electrical dorsal horn activity.

Previous studies in anaesthetized mice, using two-photon imaging and multi-cell bolus loading of non-selective cell permeant calcium indicator^28^,^31^, showed that presumed lumbar spinal cord astrocytes can exhibit slow spontaneous calcium oscillations of several seconds duration^28^. Calcium transients in response to hind paw stimulation, presumably measured in somatic regions, were also reported^31^.

We demonstrate in awake mice that, in the absence of sensory stimulation, astrocyte process activity is infrequent and unsynchronized (Fig. 5a,c). Calcium transient frequency depended on behavioural state (Fig. 5c) and was elevated in response to sensory stimulation (Fig. 5d). Sensory information was represented differently in dorsal horn astrocytes compared with neurons, based on transients measured in astrocyte processes and neuronal somata. For low- and medium-amplitude stimuli, we found little correlation between the duration of individual calcium transients in astrocyte processes and pinch duration (Fig. 5e,f) or between calcium transient amplitude in astrocyte processes and pinch pressure (Fig. 5d). However, large-scale events were evoked primarily by high-amplitude stimuli. General anaesthesia markedly suppressed astrocyte calcium transient frequency and eliminated large-scale events (Figs 5c–d and 6c).

Similar to cortical astrocytes^32^, calcium transient frequency in dorsal horn astrocytes was higher in processes compared with somatic regions (Supplementary Movie 3). Dorsal horn astrocytes showed different forms of calcium excitation (Figs 5a and 6a,b; Supplementary Movie 3), including large-scale events. However, unlike large-scale calcium transients described for cortex^4^,^22^,^23^, locomotion alone did not evoke large-scale coordinated calcium excitation in dorsal horn astrocytes (Figs 5a and 6a,c), suggesting regional differences in neuron-astrocyte communication or receptor expression. Large-scale events in dorsal horn astrocytes were seen in response to intense sensory stimulation. However, their duration and the duration of their constituent calcium transients were remarkably short compared with cortical events (Fig. 6a,b,e; Supplementary Movie 3)^4^,^22^,^23^, indicating differences in astrocyte coupling or intracellular calcium handling. The long latency of large-scale events (Fig. 6f) points to a supraspinal trigger that may be linked to stimulus intensity-dependent recruitment of nociceptive fibres.

Future studies are needed to determine how activity patterns in different neuronal compartments or types of genetically defined sensory neurons^17^,^33^,^34^ relate to calcium excitation in subsets of spinal cord astrocytes and their compartments^12^,^35^, and how in turn astrocyte calcium activity may influence sensory or pain processing in the spinal cord and brain.

One-photon imaging provides access to superficial spinal cord regions involved in sensory processing. Given that repeated calcium imaging is feasible (Supplementary Fig. 6) this together with existing mouse models of disease^36^ may allow the study of how cellular activity patterns contribute to disease, and how treatments can control aberrant activity. Optical recordings from deeper regions may be possible using longer wavelength indicators or implantation of micro-optics^3^,^37^,^38^. Optical access to regions beyond those accessible with one-photon microscopy without physical tissue penetration is already possible with multi-photon microscopy^28^. However, even latest multi-photon approaches^37^,^39^ may not be able to reach ventral motor regions or with high enough signal-to-noise ratio to monitor fast neuronal dynamics because spinal grey matter is largely encased by highly and broadband reflective white matter^40^.

Multi-photon imaging generally requires animal restraint, which can limit or alter animal behaviour^1^. At the same time, focally restraining animals both at the level of the spinal cord and head, or combining miniaturized microscope-enabled spinal cord with head-restrained recordings presents unique opportunities for concomitant imaging of anatomically connected spinal cord and brain regions. This might enable study of how spinal circuit processing shapes sensory perception or how the brain exerts supraspinal control at the cellular level. Activation of any peripheral region or primary sensory afferent in freely behaving mice can in principle be achieved using opto- or chemogenetic approaches^41^,^42^,^43^.

In summary, by producing a dynamic picture of the functioning spinal cord our imaging approaches and their future extensions promise to further our understanding of the computations underlying normal spinal cord processes, and the pathologic changes provoked by spinal cord disorders.

## Methods

### Animal subjects

All procedures were performed in accordance with the guidelines of the National Institutes of Health and were approved by the Institutional Animal Care and Use Committee at the Salk Institute. Mouse strains used in this study included wild-type C57BL6/J (Jackson Laboratories) and transgenic mice (Thy1-eYFP-H, stock #003782, Jackson Laboratories; SOM-Cre x CAG(ROSA26)-flex-tdTomato, Goulding laboratory, Salk Institute). Mice were typically group-housed at ∼22 °C and provided with bedding and nesting material. Both male and (typically) female mice were used with a typical age of 7–10 weeks at the time of imaging (5–7 weeks at the time of stereotactic injection, if performed).

### Stereotactic injections

Thin-wall glass pipettes were pulled on a Sutter Flaming/Brown micropipette puller (model P-97). Pipette tips were carefully cut at an acute angle under × 10 magnification using sterile techniques. Tip diameters were typically ∼15–20 μm. Pipettes that did not result with sharp bevels nor had larger tip diameters were discarded. Millimetre tick marks were made on each pulled needle to allow measurement of virus volume injected into the spinal cord.

Mice were anaesthetized with isoflurane (4% for induction; ∼1.5% during surgery) and positioned in a computer-assisted stereotactic system with digital coordinate readout and atlas targeting (Leica Angle Two). Body temperature was maintained at 36–37 °C with a d.c. temperature controller, and ophthalmic ointment was used to prevent eyes from drying. A small amount of depilator cream (Nair) was used to thoroughly remove hair over dorsal areas of the spinal cord at and around the L4–L5 vertebra level. Skin was cleaned and sterilized with 70% ethanol and betadine. Using surgical scissors, a small (∼10 mm) incision was made along the midline. Fascia connecting the skin to the underlying muscle were removed with forceps. Skin was held back by retractors, creating an ∼25 × 38mm exposed area. Using blunt dissection, lateral edges of the spinal column were isolated from connective tissue and muscle. Tissue from the vertebra of interest (L4–L5), and one vertebra rostral and caudal to the site of spinal cord exposure was removed with forceps. The spine was then stabilized using Cunningham vertebral clamps and any remaining connective tissue on top of the exposed vertebrae removed with a fine spatula. Using a small sterile needle a ∼0.3 mm opening was made in the tissue overlying the designated injection site between the L4 and L5 vertebra. Then, a drop of virus was carefully pipetted onto parafilm (∼1–2 μl) for filling the pulled injection needle with the desired volume. Once loaded with sufficient volume, the injection needle was slowly lowered into the spinal cord until the target depth was reached (typically 150–200 μm below the dura). Manual pressure was applied using a 30-ml syringe connected by shrink tubing and 0.6–1.0 μl of virus was slowly injected over a period of ∼5–10 min. Viruses included AAV9-CaMKII-GCaMP6f (Penn Vector Core; titre, 1.14 × 10^13^ GC per ml; dilution, 1:5; volume, 0.6 μl) and AAV5-GfaABC1D-GCaMP6f (Penn Vector Core; titre, 3.31 × 10^13^ GC per ml; no dilution; volume, 1.0 μl). Once desired volume of virus was injected, the syringe's pressure valve was locked and position maintained for ∼10 min to allow virus to spread and to avoid backflow upon needle retraction. Following the injection, vertebral clamps were removed and paraspinous muscle approximated over the entry site of the spinal cord. Mice were sutured along the skin incision, given subcutaneous Buprenex SR (0.5 mg kg^−1^) and allowed to recover before placement in their home cage.

### Live-animal surgery

Spinal imaging chamber and head plate implantation were performed as previously described^13^,^44^. Animals that received both implants underwent respective surgeries on the same day. Laminectomies (typically 2.0 × 4.0-mm wide in medio-lateral and rostra-caudal direction, respectively) were performed at the L4–L5 vertebra level, corresponding to sacral spinal cord. The dura mater overlying the spinal cord was typically kept intact, but removed for repeated imaging (Supplementary Fig. 6). To minimize tissue movement it was critical to place the imaging chamber as close to the exposed spinal cord as possible. To achieve this, we modified a previously published design^13^. Our chamber included a central opening of 11.2 × 5.1 mm with chamfered edges, which improved mechanical stability and reduced mechanical pressure on the spine. Optical windows were sealed with a custom-cut #0 coverslip. Spinal windows were performed either immediately before imaging (for Thy1-eYFP-H and SOM-tdTomato mice) or 2–3 weeks after virus injection (14 days for AAV9-CaMKII-GCaMP6f or 21 days for AAV5-GfaABC1D-GCaMP6f). Buprenex SR (0.5 mg kg^−1^) was given to minimize postoperative pain and hypersensitivity^45^.

### *In vivo* imaging

For imaging in freely behaving mice, we used miniaturized one-photon microscopes custom made at the Salk Institute. Compared with our previously published versions^3^,^5^, these microscopes provided a larger FOV (720 μm × 960 μm), improved lateral resolution (≤4–6 μm in central regions of the FOV; using an Edmund Optics NT45-549 drum lens, GRINTECH GT-IFRL-200-confD-50 GRIN lens, and Edmund Optics NT45-207 achromatic lens; Supplementary Fig. 2; Supplementary Movie 1), higher sensitivity (6.7 V per lux-sec) and frame rate (45 f.p.s. at full resolution; using an Aptina MT9M024 complementary metal-oxide semiconductor image sensor, custom-made headboard and Aptina AGB1N0CS interface PCB). For imaging of GCaMP6f- or eYFP-expressing cells, the microscope was equipped with a miniature blue light-emitting diode (LED; LXML-PB01-0023, Philips Luxeon Rebel), single-band T495lpxr beam splitter, ET480/40x excitation filter and ET535/50-m emission filter (Chroma). For imaging of tdTomato-expressing cells, we used a miniature yellow LED (LXML-PX02-0000; Philips Luxeon Rebel), dual-band 59004bs beam splitter, 59004x excitation filter and 59004m emission filter (Chroma). A custom-made LED driver was used to correct for LED intensity drift. Typical average power used for imaging was 100-125 μW mm^−2^ or 150–175 μW mm^−2^ for eYFP / tdTomato- or GCaMP6f-expressing cells, respectively. Typical image resolution was 1,280 × 960 pixels. In a typical imaging session between 80 and 100 recordings were taken, each lasting approximately 1–2 min. No signs of phototoxicity, such as a gradual increase in baseline fluorescence, lasting changes in spike rate or blebbing of labelled cells were apparent in our recordings.

For imaging in awake mobile mice, restrained at the spinal cord and head level, we used an upright two-photon microscope (Sutter Instrument Company) equipped with an 8-kHz resonant scanner (Cambridge Technology, Inc.), a pulsed femtosecond Ti:Sapphire laser (Chameleon Vision II, Coherent), a T565LPXR beam splitter (Chroma), ET525/70M and ET605/70M emission filters (Chroma), two GaAsP photomultiplier tubes (H10770PA-40 MOD; Hamamatsu) and either a × 20 1.0 numerical aperture (NA; XLUMPlanFLN; Olympus) or × 16 0.8 NA water-immersion objective (CFI75; Nikon). Custom modifications to this microscope included the integration of an adaptive focus control unit (WDI Wise Device Inc.), allowing real-time measurement and correction of axial distance changes between microscope objective and optical window with sub-micrometre precision^18^. Typical average power used for imaging lamina I and II of Thy1-eYFP-H or SOM-tdTomato mice was 10–25 mW. Twenty to 30 mW was used for imaging of AAV-injected animals expressing GCaMP6f in either dorsal horn neurons or astrocytes. Image resolution was 256 × 512 pixels (frame rate, 61.8 f.p.s.) or 512 × 512 pixels (frame rate, 30.9 f.p.s.).

Imaging of the same dorsal horn cells with two- and miniaturized one-photon microscopy on a given day (Supplementary Figs 1 and 5), and repeated imaging of the same cells over multiple days (Supplementary Fig. 6) was achieved by precisely aligning the animal under the microscope using characteristic surface blood vessels as reference structures, similar to our previous work^46^.

### *In vivo* image data processing and analysis

One-photon images from freely behaving mice were cropped to fluorescently labelled, central FOV areas. Within-frame distortions in cropped images were typically small and therefore not corrected. Full-frame lateral motion artefacts were corrected using *TurboReg*^15^ or manually. Motion-corrected calcium imaging data were analysed using custom MATLAB software based on principal and independent component analysis (PCA/ICA)^20^, which automatically computes ROI spatial filters and minimizes signal crosstalk. PCA/ICA analysis was supplemented by manual ROI analysis to include cells that were apparently missed by the algorithm. For manually selected cells to be included in analysis, these cells had to exhibit a ≥50% Δ*F*/*F* calcium transient at least once during the entire imaging session, which included presentations of different stimuli or stimulus intensities under anaesthetized and awake conditions. ROI analysis was performed using ImageJ.

Two-photon imaging data from awake mobile mice were corrected for lateral motion artefacts using *TurboReg*^15^. Motion-corrected calcium imaging data were analysed using MATLAB-based PCA/ICA or ImageJ-based ROI analysis, as described above.

To identify calcium transients within extracted GCaMP6f activity traces, we computationally searched for local maxima with (a) a peak amplitude more than 2 s.d.'s (2*σ*) from the trace's baseline, (b) ≥0.2 s surrounding the peak having a minimum intensity >2*σ* and (c) ≥0.2 s separation between transients. For astrocyte calcium excitations above 2*σ* with two or more peaks, we counted each peak as separate transient if the amplitude between peaks fell below the half maximum of the lowest peak for ≥0.2 s. Calcium transient occurrence was set to the temporal midpoint in the rise to peak fluorescence from the most recent trough, approximating a time midway in the corresponding calcium spike.

For neurons, calcium transients were considered stimulus evoked if they occurred within 1 s after stimulus delivery and were not associated with measureable locomotor activity of the animal, as determined by behavioural video. Calcium transients were considered movement evoked if they occurred in the absence of a deliberate stimulus and within 400 ms of measureable bodily movement.

For astrocytes, calcium transients were considered stimulus evoked if they occurred during stimulus delivery. Calcium transients were considered movement evoked if they occurred in the absence of a deliberate stimulus and during running, as determined by behavioural video or encoder data. Calcium transients were considered a large-scale coordinated event if (a) at least seven ROIs within the FOV had a peak amplitude more than 2 s.d.'s (2*σ*) from the trace's baseline and (b) during a ±1.5 s time window surrounding this event at least 15 ROIs had a mean intensity >2*σ* for ≥0.2 s. Large-scale events were considered stimulus evoked if they occurred within 7 s after stimulus delivery, and running evoked if they occurred in the absence of a deliberate stimulus and within 7 s after running onset. When stimulus and locomotor activity coincided, we calculated the latency to onset and offset to determine transients' functional relationship (Fig. 6f,g).

Behavioural video data from freely moving mice were manually scored for periods of rest, movement, grooming or other forms of animal activity. Encoder data from restrained mice on a spherical treadmill were quantified as previously described^4^,^20^.

To determine imaging depth and spatial resolution of our miniaturized one-photon microscopes, we recorded from the same spinal cord region in fluorescent reporter (Thy1-eYFP-H or SOM-tdTomato) mice using two- and miniaturized one-photon microscopy. Maximum imaging depth was defined as the sub-dural focal depth up to which the same cellular structures could be identified in fluorescence images acquired with both imaging modalities (Supplementary Fig. 1). Spatial resolution was determined by comparing full width at half maximum (FWHM) from line profiles through the same cellular structures recorded with both imaging modalities (Supplementary Fig. 2).

Axial tissue movement was estimated by first recording two- or one-photon images at different sub-dural focal positions in the anaesthetized animal (typically 40 images acquired at 1 μm axial spacing). Focal shifts in optical recordings from the behaving animal were then determined by comparing cellular structures (for example, FWHM of cell bodies or processes) in the time-lapse images with the same structures in the reference *z*-stack acquired under anaesthesia.

### Analogue and video data processing and analysis

Cutaneous stimuli were applied to the base of the animal's tail (at ∼5-mm distance from the animal's dense coat hair). Pressure stimuli (30, 70, 130, 220, 400, 600 or 800 g) were applied using a rodent pincher system (2450, IITC Life Science, Inc.). The order in which these stimuli were applied was randomized. Inter-stimulus interval was typically ≥60 s. On the basis of the previous work^33^,^47^,^48^, three pressure categories were defined for analysis: low- (*P*=30–70 g), medium- (*P*=130–220 g) and high-amplitude pressure (*P*=400–800 g). Pressure sensor output was recorded using MCS software (Sutter Instrument Company; sampling rate, 1 kHz). Brief air puff stimuli were applied using a compressed air canister with its nozzle pointing at the target area and away from coat hair (nozzle tip-to-target area distance, ∼15 mm). Air puff application and mouse behaviour were recorded on video camera (Stingray F-033, Allied Vision Technologies; 30 or 60 f.p.s.). For mice focally restrained on a spherical treadmill running speed was also tracked using an optical encoder (E7PD-720-118, US Digital). The order in which different stimuli were applied was randomized. Inter-stimulus interval was typically ≥60 s.

To synchronize one- or two-photon fluorescence imaging data with video data, we placed a 680- or 870-nm LED, triggered from the miniaturized microscope's light source or two-photon microscope's shutter TTL signal, within the video camera's FOV. Video data were cropped to the LED-on period. One-photon imaging and analogue data were synchronized by recording the on–off TTL signal of the miniaturized microscope's light source together with the analogue output signal from the pressure sensor in MCS. The pincher trace was then cropped to the light source-on period. Two-photon imaging and pressure sensor and/or encoder data were synchronously recorded in MCS.

Pressure sensor data were recorded at 4 Hz (limited by the pressure metre and limiting measurement accuracy of response latency in Fig. 3d). Pressure stimulus onset was defined as the point at which the smoothed pressure trace exceeded *P*=10 g. This definition was used to calculate calcium transient latency shown in Figs 3d and 6f. Pressure traces were quantified with respect to stimulus amplitude and duration using custom MATLAB (Mathworks) routines. Only data for which peak stimulus amplitude remained stable (within 10, 30 or 60 g for low-, medium- or high-amplitude stimuli, respectively) for at least 50% of the pinch duration, defined as FWHM, was included in analysis (Figs 3e,f and 5e,f). Likewise, only data for which pinch duration were similar (typically within ±1.25 s of the target duration; within ±20% of the short target duration shown in Fig. 4g) for a given peak stimulus amplitude was included in analysis (Fig. 3g–i; Fig 4c,d,g–h; Fig. 5d; Fig. 6c–g).

Encoder data were recorded at 1 kHz. Encoder traces were smoothed using a sliding average (window size, 0.4 s). Locomotion onset or offset was defined as the point at which the smoothed running speed exceeded or fell below 10 mm s^−1^. Encoder traces were analysed with respect to maximum and average running speed, running duration and frequency of running.

Supplementary Movies were generated using Adobe Premier.

### Behavioural data processing and analysis

To quantify locomotor performance of freely behaving mice, animals were placed on a motorized linear treadmill (Exer Gait XL, Columbus Instruments). Mouse behaviour was recorded using two video cameras, one monitoring the animal's footsteps from below the translucent treadmill belt (at 100 f.p.s.) and the other monitoring the animal's vertical activity (at 60 f.p.s.) through a transparent side wall parallel to the belt's movement direction. Hind limb stride length was extracted from footstep recordings using TreadScan (Clever Sys Inc.). Maximum running speed was defined as the belt speed up to which animals were able to avoid end wall collisions during the 1–2 min measurement period, during which belt speed was gradually and manually increased. Mice were habituated to the linear treadmill before recordings.

To quantify locomotor performance of awake mobile mice, focally restrained animals were placed on a spherical treadmill for ∼5 min for each restraint type (head restraint, spinal cord restraint or combined spinal cord and head restraint). The order in which animals were subjected to the three different restraint types was randomized. Animals were allowed to rest for at least 10 min between recordings for each restraint type (10 sessions over 3 consecutive days). Collected encoder data were analysed with regard to rest and running periods, maximum and average running speed using custom MATLAB (Mathworks) routines. Mice were habituated to focal restraint on the spherical treadmill for 5 consecutive days before recordings.

### Immunofluorescence

Mice were killed in their home cage at different time points after viral vector injection, spinal imaging chamber implantation or *in vivo* imaging using CO_2_ asphyxiation at a 20% fill rate, in accordance with Institutional Animal Care and Use Committee guidelines. Animals were then quickly transcardially perfused with 10% sucrose followed by 4% paraformaldehyde. Spinal cord tissue between the L1 and L5 vertebra level was carefully extracted and allowed to incubate in 4% paraformaldehyde overnight at 8 °C. Fixed tissue was thoroughly washed on a plate shaker with 1 × PBS three times over ∼1 h.

Perfused and PBS-washed tissue was then sectioned at 50 μm using a Leica VT1000s model vibratome. Immunostaining was performed on floating coronal sections using standard techniques. Primary antibodies included GFAP 1:250 (Millipore Cat. #MAB3402). Secondary antibodies (1:100) included Alexa Fluor 633 goat anti-mouse (Life Technologies Cat. #A21052).

Confocal imaging of stained tissue sections was performed on a Zeiss LSM 780. Two channel tiled *z*-stacks were acquired to produce images of whole-tissue sections (laser lines: 488 nm, 633 nm). Image size was 1,024 × 1,024 pixels stitched into 3–5 × 3–5 tiles (frame scanning; pixel dwell time, 1.58 μs; average, 2 frames). Images were taken with an Olympus × 20 0.8 NA air-matched objective.

Images were processed and analysed using ImageJ software.

### Statistical analysis

Data were analysed and plotted using MATLAB, Excel or Prism software. All data are represented as mean±s.e.m. Group sample sizes were chosen based on previous studies and/or power analysis. The following convention was used to indicate *P* values: ‘NS' indicates *P*>0.05, ‘*' indicates 0.01<*P*≤0.05, ‘**' indicates 0.001<*P*≤0.01, ‘***' indicates 0.0001<*P*≤0.001, and ‘****' indicates *P*≤0.0001.

## Additional information

**How to cite this article:** Sekiguchi, K. J. *et al*. Imaging large-scale cellular activity in spinal cord of freely behaving mice. *Nat. Commun.* 7:11450 doi: 10.1038/ncomms11450 (2016).

## Supplementary Material

Supplementary Information Supplementary Figures 1-6

Supplementary Movie 1 Pinch-evoked calcium activity in dorsal horn neurons of an anesthetized mouse

Supplementary Movie 2 Hindquarter grooming-evoked calcium activity in dorsal horn neurons of a freely behaving mouse

Supplementary Movie 3 Ongoing and pinch-evoked calcium activity in dorsal horn astrocytes of an awake mobile mouse
