# Supplementary material for: Imaging large-scale cellular activity in spinal cord of freely behaving mice
Source: Nat Commun. 2016 Apr 28;7:11450. doi: 10.1038/ncomms11450 (PMC4853475; doi:10.1038/ncomms11450)
Supplement: Supplementary Information — Supplementary Figures 1-6 [file ncomms11450-s1.pdf]

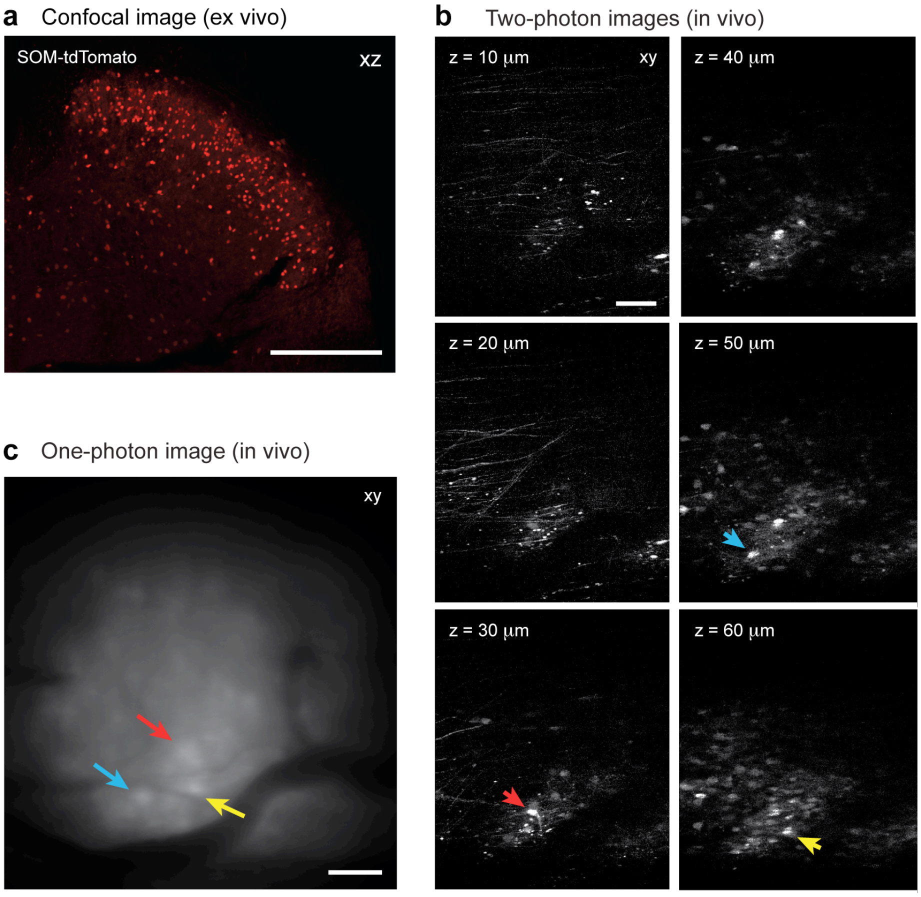

Supplementary Figure 1 | Dorsal horn imaging depth with miniaturized one-photon microscopy. (**a**) Confocal image showing dorsal horn cells in a coronal (xz) spinal cord section from a SOM-tdTomato mouse. tdTomato expression in this mouse line is restricted to somatostatin (SOM) lineage neurons, whose cell bodies are located primarily in lamina II and below. Scale bar, 200 μm. (**b**) Two-photon images from a z-stack showing tdTomato-expressing cells in an anesthetized SOM-tdTomato mouse. Focal depth (z) below the dura is indicated in the transverse (xy) images. Scale bar, 50 μm. (**c**) One-photon image taken with a miniaturized microscope from the same dorsal horn region shown in **b**. Three tdTomato-expressing cells that appear in both one- and two-photon images are indicated (blue, red, and yellow arrows). The one-photon volume image captures cells located at different focal depth, allowing their simultaneous recording. Scale bar, 50 μm.

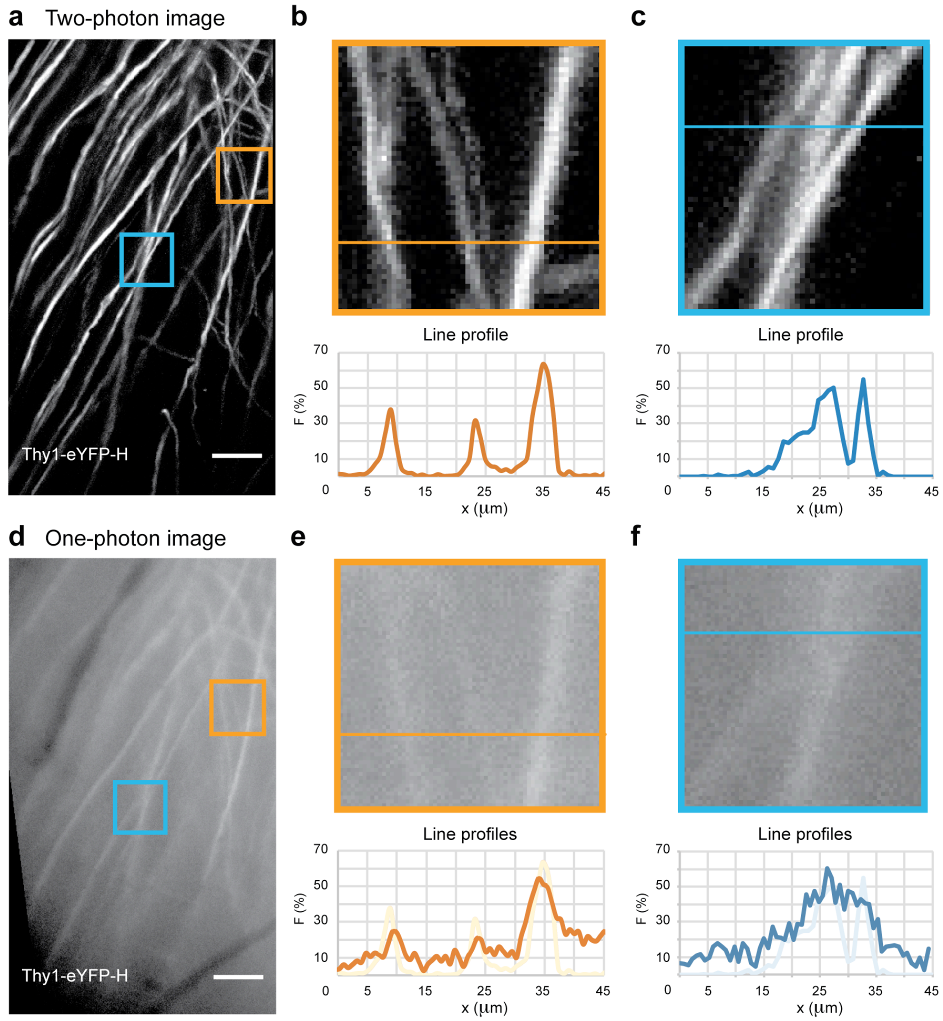

Supplementary Figure 2 | Spatial resolution with miniaturized one-photon microscopy. (**a**) Maximum-intensity projection image from a two-photon z-stack (depth, 3-27 μm below the dura; axial spacing, 1 μm; frame average, 8) showing sparsely labeled axons in dorsal spinal cord of an anesthetized Thy1-eYFP-H mouse (same image as in **Fig. 2d**). Two regions of interest (ROIs; orange and blue) are indicated. Scale bar, 50 μm. (**b**) *Top*, orange boxed region in **a** shown at higher magnification. *Bottom*, relative fluorescence intensity profile along the orange horizontal line indicated in the magnified image. (**c**) *Top*, blue boxed region in **a** shown at higher magnification. *Bottom*, relative fluorescence intensity profile along the blue horizontal line indicated in the magnified image. (**d**) One-photon volume image (same image as in **Fig. 1d**) showing the same spinal cord region as in **a**. The same two ROIs as in **b** were analyzed. Scale bar, 50 μm. (**e**) *Top*, orange boxed region in **d** shown at higher magnification. *Bottom*, relative fluorescence intensity profile along the orange horizontal line indicated in the magnified image. The corresponding two-photon line profile is overlaid in light orange. (**f**) *Top*, blue boxed region in **d** shown at higher magnification. *Bottom*, relative fluorescence intensity profile along the blue horizontal line indicated in the magnified image. The corresponding two-photon line profile is overlaid in light blue. Closely spaced axons or axon bundles cannot be resolved with miniaturized one-photon microscopy.

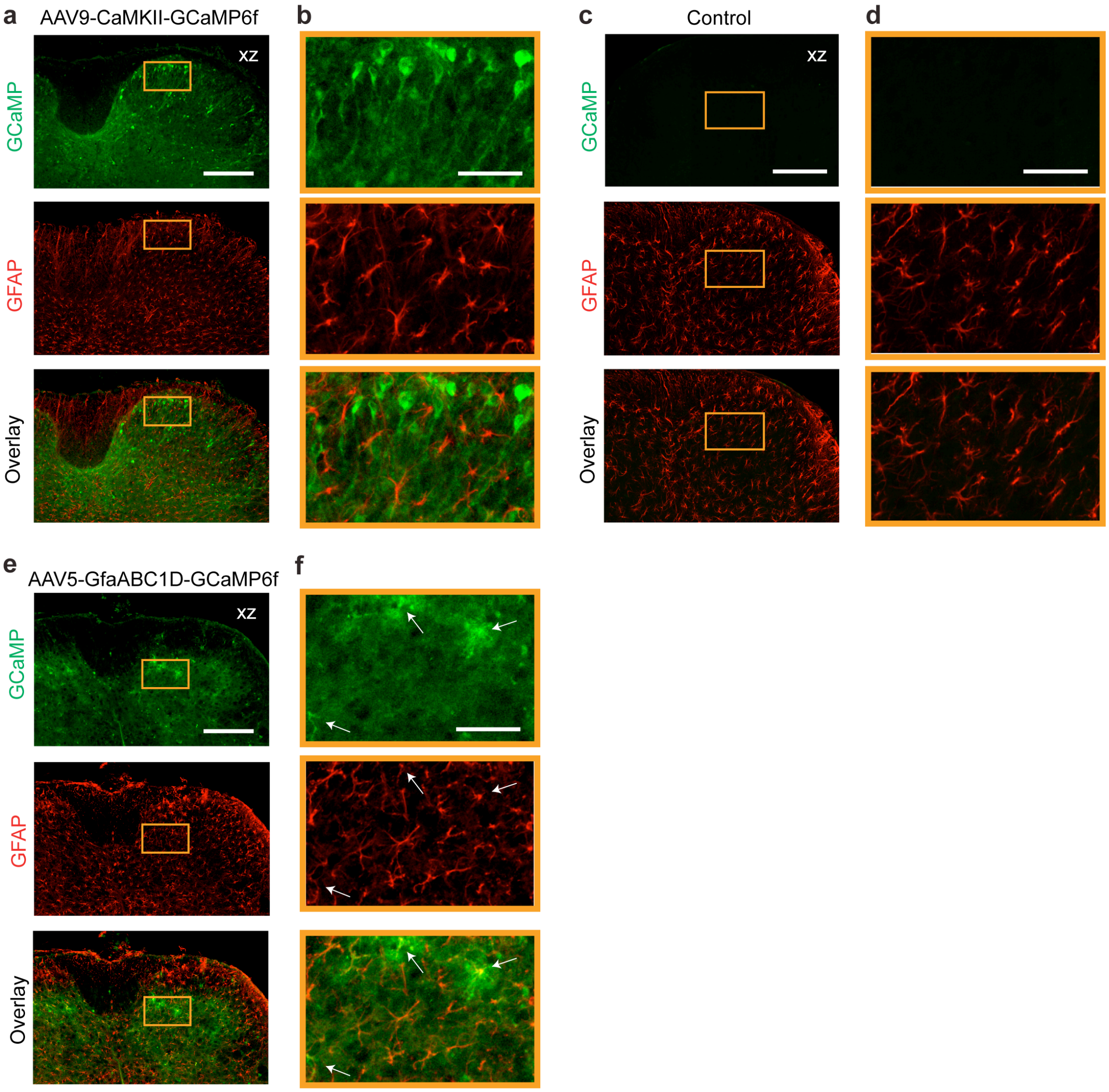

Supplementary Figure 3 | AAV-mediated labeling of spinal cord neurons and astrocytes. (**a**) *Top*, confocal image showing GCaMP6-transduced dorsal horn cells in a coronal (xz) lumbar (L4-L5 vertebra) level section from a wild type mouse 14 days after AAV9-CaMKII-GCaMP6f injection, and immediately after spinal cord window implantation and *in vivo* imaging. *Center*, glial fibrillary acidic protein (GFAP) co-staining. *Bottom*, overlay image. Scale bar, 150 μm. (**b**) Magnified images of the boxed regions (orange) shown in **a**. Scale bar, 50 μm. (**c**) Confocal images from a coronal L1-L2 vertebra level control section obtained from the same mouse shown in **a**. *Top*, image showing lack of GCaMP6-transduction. *Center*, GFAP staining. *Bottom*, overlay image. Scale bar, 150 μm. (**d**) Magnified images of the boxed regions (orange) shown in **c**. Scale bar, 50 μm. (**e**) Confocal images from a wild type mouse 21 days after AAV5-GfaABC1D-GCaMP6f injection, and immediately after spinal cord window implantation and *in vivo* imaging. GCaMP6 staining (green) and GFAP co-staining (red) are shown atop the overlay image. Scale bar, 150 μm. (**f**) Magnified images of the boxed regions (orange) shown in **e**. A subset of co-stained cells is indicated. Scale bar, 50 μm.

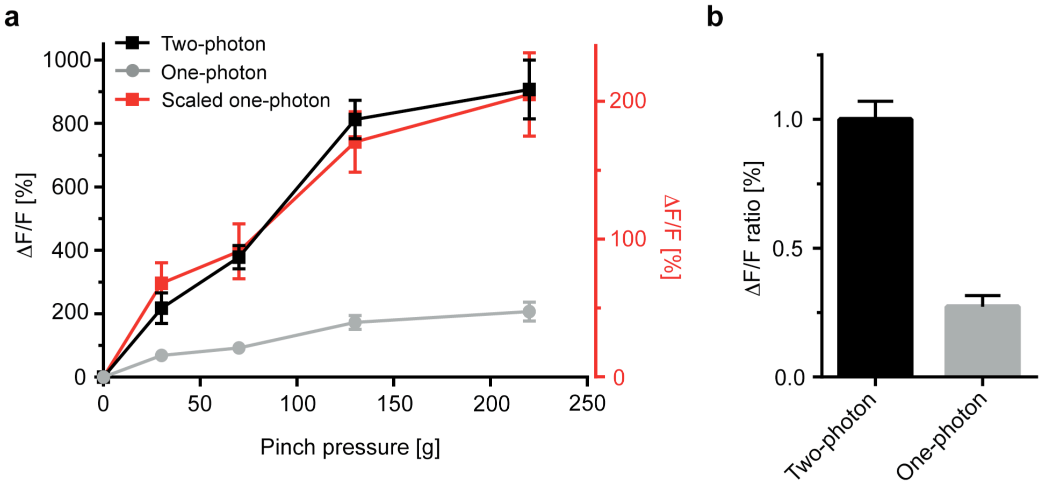

Supplementary Figure 4 | Sensitivity with miniaturized one-photon microscopy. (**a**) Average pinch-evoked calcium transient amplitude as a function of pinch pressure for a representative dorsal horn neuron in an anesthetized wild type mouse injected with AAV9-CaMKII-GCaMP6f. The same cell was measured with two- (black; average power, 20 mW) and miniaturized one-photon microscopy (gray; average power, 158 μW per mm^2). While both imaging modalities yield the same functional relationship (red, scaled one-photon trace), small differences in pressure amplitude can be more readily decoded from calcium transients measured with two-photon microscopy in focally restrained mice. (**b**) Population data showing relative calcium transient amplitude of miniaturized one-photon (gray) compared to two-photon microscopy (black). Data are represented as mean ± SEM.

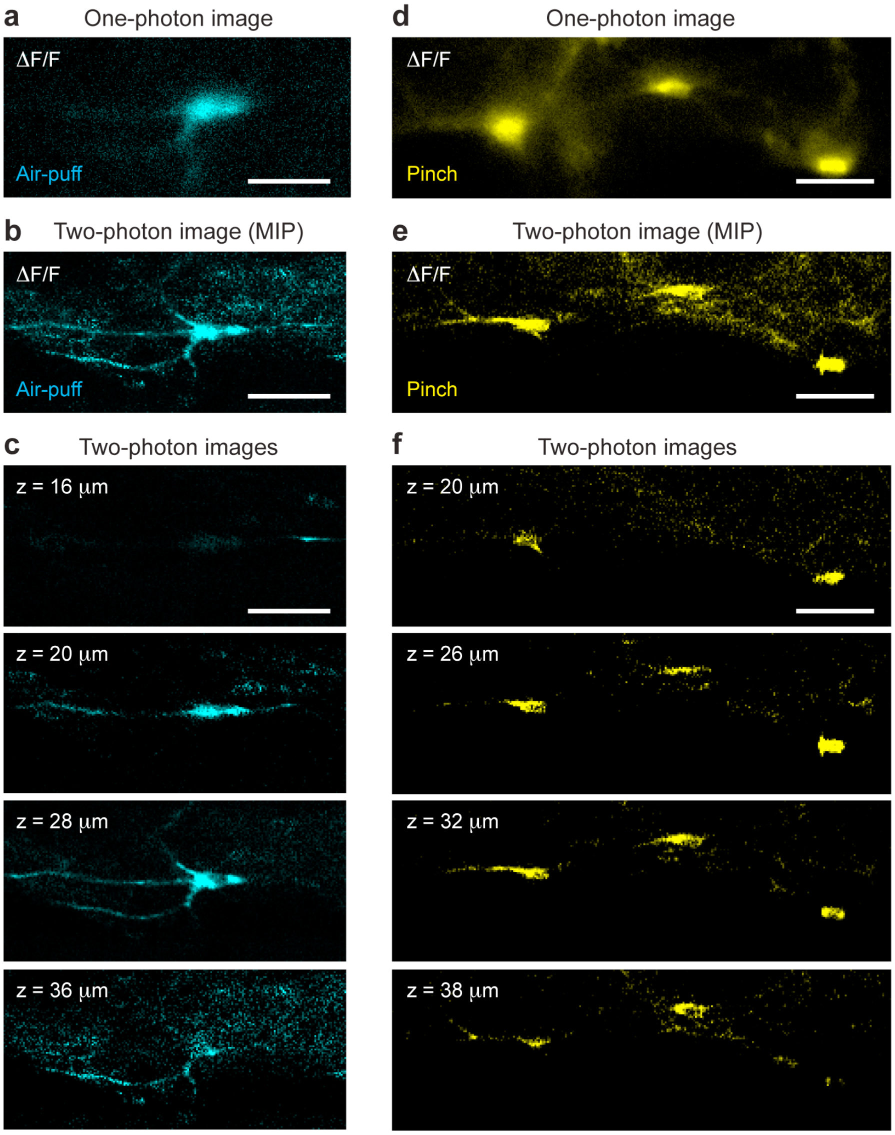

Supplementary Figure 5 | Depth determination of peripheral stimulus-responsive dorsal horn neurons recorded with miniaturized one-photon microscopy. (**a**) Fluorescence image showing a dorsal horn neuron in an AAV9-CaMKII-GCaMP6f-injected wild type mouse activated by air puff (blue). Data was recorded in an unrestrained awake mouse using miniaturized one-photon microscopy. Scale bar, 30 μm. (**b**) Maximum-intensity projection (MIP) image from a two-photon z-stack (depth, 16-36 μm below the dura; axial spacing, 2 μm; frame average, 8) showing the same dorsal horn neuron as in **a**. Data was recorded in the awake focally restrained mouse using two-photon microscopy. Similar air puff application was used as during the corresponding one-photon recording. Scale bar, 30 μm. (**c**) Individual two-photon images from the z-stack shown in **b**. Focal depth (z) below the dura is indicated in each image. Scale bar, 30 μm. (**d**) Fluorescence image showing dorsal horn neurons in an AAV9-CaMKII-GCaMP6f-injected wild type mouse activated by pinch (yellow; P = 220 g). Data was recorded in an unrestrained awake mouse using miniaturized one-photon microscopy. Scale bar, 30 μm. (**e**) Maximum-intensity projection (MIP) image from a two-photon z-stack (depth, 20-40 μm below the dura; axial spacing, 2 μm; frame average, 8) showing the same dorsal horn neurons as in **d**. Data was recorded in the awake focally restrained mouse using two-photon microscopy. Comparable pinch parameters were used as during the corresponding one-photon recording. Scale bar, 30 μm. (**f**) Individual two-photon images from the z-stack shown in **e**. Focal depth (z) below the dura is indicated in each image. Scale bar, 30 μm.

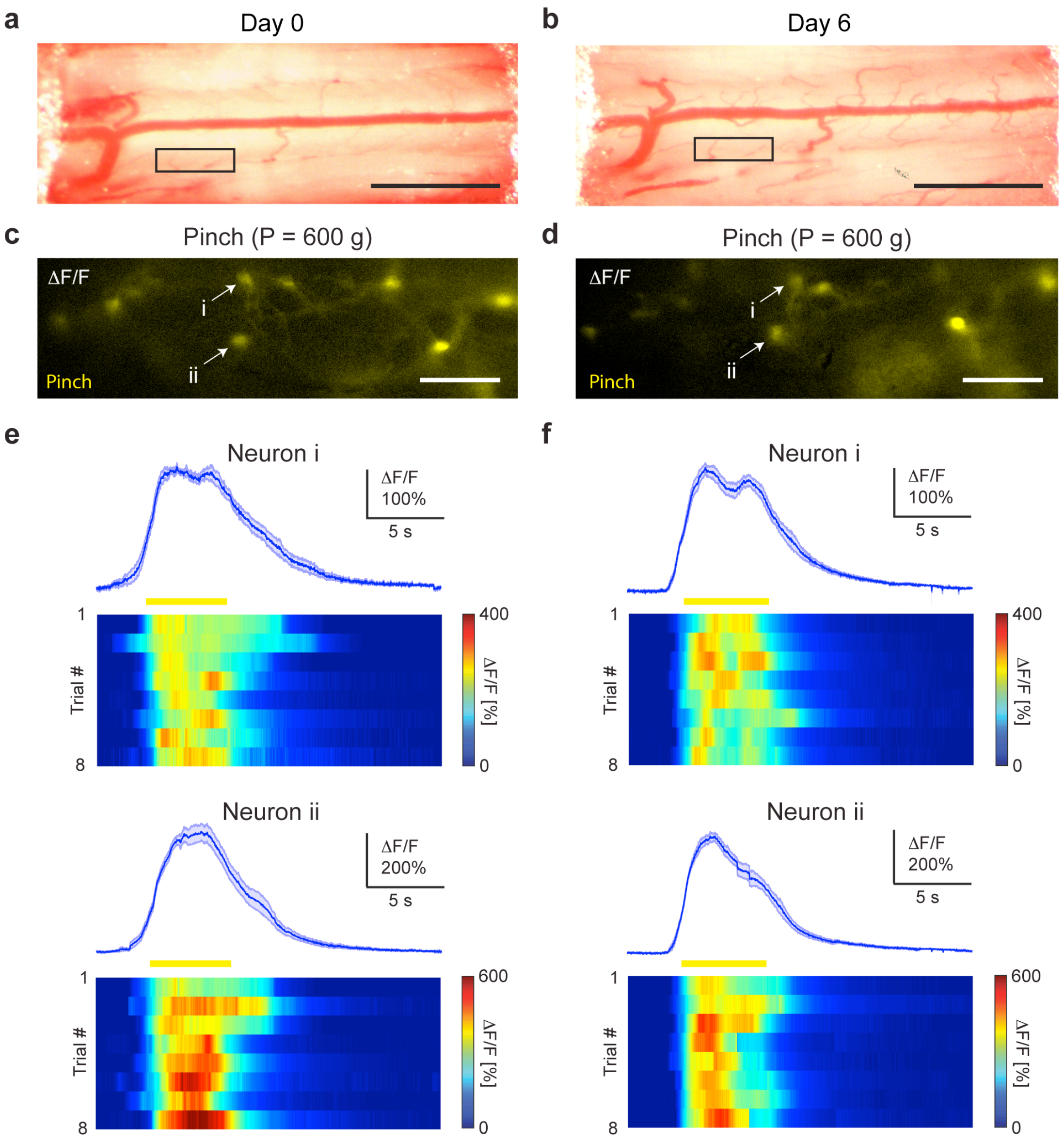

Supplementary Figure 6 | Repeated imaging of pinch-evoked calcium activity from dorsal horn neurons. (**a**) Top view onto a lumbar spinal cord window implanted in a wild type mouse that was injected with AAV9-CaMKII-GCaMP6f. The image was taken on the day of surgical preparation (day 0). Virus injection was performed 14 days earlier. Scale bar, 1 mm. (**b**) Top view onto the same spinal cord window shown in **a** 6 days after surgical preparation. The window remained transparent, allowing repeated imaging. Scale bar, 1 mm. (**c**) Fluorescence image showing dorsal horn neurons activated by pinch under isoflurane anesthesia at day 0 (yellow; P = 600 g). Data was recorded from the boxed region indicated in **a** using miniaturized one-photon microscopy. Two example neurons are indicated. Scale bar, 100 μm. (**d**) Fluorescence image showing pinch-responsive dorsal horn neurons from the same region as in **c** 6 days after surgical preparation. Comparable pinch parameters were used, and the same two neurons as in **c** are indicated. Many of the same neurons responded similarly to pinch across days. Scale bar, 100 μm. (**e**) Average calcium transient (blue trace, top) and single trial responses (bottom) to pinch at day 0 for the two neurons indicated in **c**. Yellow horizontal bars indicate peripheral stimulus duration. Note the distinct average response amplitude and kinetics of neurons i and ii. (**f**) Average calcium transient (blue trace, top) and single trial responses (bottom) to pinch at day 6 for the same two neurons shown in **e**. Yellow horizontal bars indicate peripheral stimulus duration. Average response amplitude and kinetics for neurons i and ii was similar across days using comparable pinch parameters. Data are represented at mean ± SEM.
